# Stand Up to Stand Out: Natural Dietary Polyphenols Curcumin, Resveratrol, and Gossypol as Potential Therapeutic Candidates against Severe Acute Respiratory Syndrome Coronavirus 2 Infection

**DOI:** 10.3390/nu15183885

**Published:** 2023-09-06

**Authors:** Zhonglei Wang, Xian-qing Song, Wenjing Xu, Shizeng Lei, Hao Zhang, Liyan Yang

**Affiliations:** 1 Key Laboratory of Green Natural Products and Pharmaceutical Intermediates in Colleges and Universities of Shandong Province, School of Chemistry and Chemical Engineering, Qufu Normal University, Qufu 273165, China; 18263526310@163.com (W.X.); 19553752811@163.com (S.L.); 17660215359@163.com (H.Z.); 2 School of Pharmaceutical Sciences, Key Laboratory of Bioorganic Phosphorus, Chemistry & Chemical Biology (Ministry of Education), Tsinghua University, Beijing 100084, China; 3 General Surgery Department, Baoan Central Hospital, Affiliated Baoan Central Hospital of Guangdong Medical University, Shenzhen 518000, China; 4 School of Physics and Physical Engineering, Qufu Normal University, Qufu 273165, China

**Keywords:** COVID-19, SARS-CoV-2, natural dietary polyphenols, curcumin, resveratrol, gossypol, nanotechnology, lead optimization, combination therapies, broad-spectrum activities

## Abstract

The COVID-19 pandemic has stimulated collaborative drug discovery efforts in academia and the industry with the aim of developing therapies and vaccines that target SARS-CoV-2. Several novel therapies have been approved and deployed in the last three years. However, their clinical application has revealed limitations due to the rapid emergence of viral variants. Therefore, the development of next-generation SARS-CoV-2 therapeutic agents with a high potency and safety profile remains a high priority for global health. Increasing awareness of the “back to nature” approach for improving human health has prompted renewed interest in natural products, especially dietary polyphenols, as an additional therapeutic strategy to treat SARS-CoV-2 patients, owing to its good safety profile, exceptional nutritional value, health-promoting benefits (including potential antiviral properties), affordability, and availability. Herein, we describe the biological properties and pleiotropic molecular mechanisms of dietary polyphenols curcumin, resveratrol, and gossypol as inhibitors against SARS-CoV-2 and its variants as observed in in vitro and in vivo studies. Based on the advantages and disadvantages of dietary polyphenols and to obtain maximal benefits, several strategies such as nanotechnology (e.g., curcumin-incorporated nanofibrous membranes with antibacterial-antiviral ability), lead optimization (e.g., a methylated analog of curcumin), combination therapies (e.g., a specific combination of plant extracts and micronutrients), and broad-spectrum activities (e.g., gossypol broadly inhibits coronaviruses) have also been emphasized as positive factors in the facilitation of anti-SARS-CoV-2 drug development to support effective long-term pandemic management and control.

## 1. Introduction

The large-scale outbreak of coronavirus disease 2019 (COVID-19), caused by the highly transmissible and pathogenic severe acute respiratory syndrome coronavirus 2 (SARS-CoV-2), placed a significant burden on the economies and healthcare sector globally [1,2]. Remarkably, several vaccines (e.g., Comirnaty^®^, Spikevax^®^, Vaxzevria, Sinopharm^®^, and Nuvaxovid™) [3], herbal medicines (e.g., Lianhuaqingwen capsules) [4,5], and small-molecule therapies (e.g., Veklury^®^ [6], azvudine [7], VV116 [8], Lagevrio^®^ [9], and Paxlovid^®^ [10]) targeting SARS-CoV-2 were approved and deployed in the clinic in a relatively short time frame. On 5 May 2023, the World Health Organization declared the end of the global health emergency, with a final death toll of at least 20 million [11]. However, SARS-CoV-2 remains a threat as it continues to spread globally. In addition, many people also continue to suffer from non-negligible long-term effects of COVID-19 (long COVID; post-acute sequelae of COVID-19), and SARS-CoV-2 variants/subvariants (e.g., Omicron variants BA.2.75, XBB.1.5, XBB.1.16, BQ.1, CH.1.1, and FE.1) are still emerging [12,13]. Long COVID is a multi-systemic disease with differing pathology in numerous organs (more than 200 symptoms were identified) [14]. Furthermore, long COVID occurs in at least 10% of SARS-CoV-2 infections, including non-hospitalized cases (10–30%), hospitalized cases (50–70%), and vaccinated cases (10–12%) [14]. In parallel, although SARS-CoV-2 vaccination significantly reduces mortality (similar to seasonal influenza), it has little effect on the viral transmission rate, thus contributing to the burden on the healthcare sector and economies of countries globally. A conservative estimate of the annual burden of SARS-CoV-2 infection suggests that it will be twice that of previous influenza seasons [15]. Therefore, there is an urgent need for a highly effective antiviral agent.

**Figure 1:**
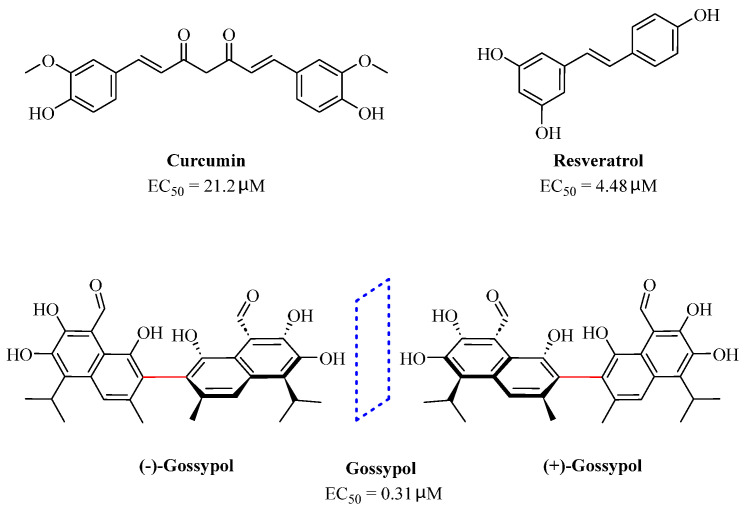
The chemical structures of curcumin, resveratrol, and gossypol. Gossypol exists as enantiomers due to the restricted rotation around the internal binaphthyl bond.

Natural dietary products have exceptional nutritional value, good safety profiles, health-promoting properties, and are abundantly available [16]. Fruits, vegetables, and spices are potential sources of dietary polyphenols that provide important benefits to human health, partly attributed to their anti-inflammatory, antioxidant, and antiviral properties [17,18,19,20]. Repurposing existing dietary polyphenols is an attractive approach for preventing or treating SARS-CoV-2 infection [21]. Numerous studies, both in in vitro and in vivo, have shown that dietary polyphenols (e.g., curcumin [22], resveratrol [23], and gossypol [24]; Figure 1) are beneficial for COVID-19 treatment via targeting of the SARS-CoV-2 papain-like protease (PL^pro^), main protease (M^pro^, 3CL^pro^), RNA-dependent RNA polymerase (RdRp), and spike (S) glycoprotein [25]. Building on our previously published work [15,26], the biological properties and pleiotropic molecular mechanisms of natural dietary polyphenols as inhibitors against SARS-CoV-2 and its variants, observed in in vitro and in vivo studies, are described to support anti-SARS-CoV-2 drug discovery and development. Nonetheless, the development of dietary polyphenol-based next-generation therapies for SARS-CoV-2 still faces multiple potential challenges. Here in this review, we discuss future representative directions, combination therapies, nanotechnology, drug delivery, lead optimization, and broad-spectrum activities of dietary polyphenols targeting SARS-CoV-2 and its variants.

## 2. Curcumin—A Turmeric-Derived Complementary Drug against COVID-19

**Figure 2:**
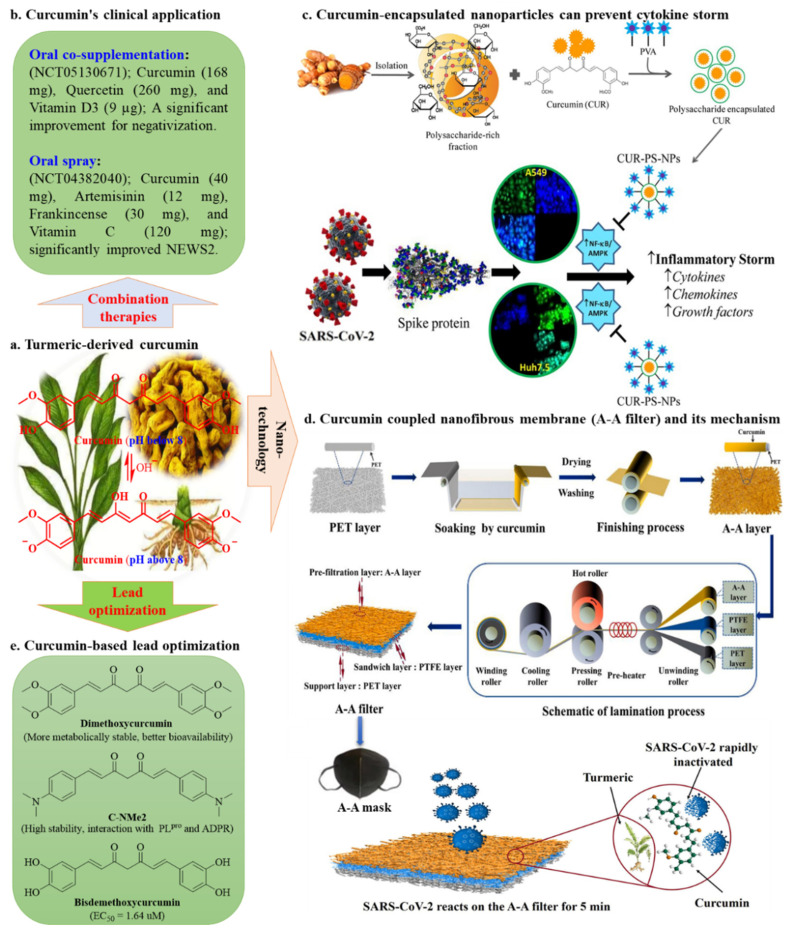
Curcumin, a turmeric-derived complementary drug, may be effective against COVID-19 with the assistance of combination therapies, nanotechnology, and lead optimization. (**a**) The chemical structure of curcumin isolated from *Curcuma longa* L. (**b**) Clinical trials of curcumin in the form of oral co-supplementation and throat spray. (**c**) Schematic representation of the CUR-PS-NP preparation process and its use in preventing lung and liver injuries associated with SARS-CoV-2 spike protein-mediated cytokine storms. (Adapted with permission from TOC and Figure 2 of Sharma et al.; ACS Appl. Bio Mater. 2022, *5*, 483–491 [57]. Copyright © 2022 American Chemical Society). (**d**) The process involved in the preparation of curcumin-incorporated nanofibrous membranes (A–A mask) and a schematic representation of the SARS-CoV-2 inactivation mechanism. (Adapted with permission [56]. Copyright © 2022 Elsevier B.V.) (**e**) Curcumin-based lead optimization.

Curcumin, a health-promoting polyphenol isolated from the dietary spice turmeric (*Curcuma longa* L.) (Figure 2a), has several well-known pharmacological properties, including antioxidant [27], anti-inflammatory [28], antifungal [29], neuroprotective [30], anticancer [31], and wound-healing effects [32]. Curcumin also has broad-spectrum antiviral activity in vitro with low-micromolar efficacy. Targeted viruses include the Zika virus (IC_50_ = 1.9 μM) [33], HIV-1 virus (IC_50_ = 12 μM) [34], and SARS-CoV (IC_50_ = 5.7 μM) [35]. Curcumin is particularly effective in treating respiratory diseases, including acute lung injury [36], pulmonary fibrosis [37], allergic asthma [38], pulmonary infections [39], and chronic obstructive pulmonary disease [40]. Furthermore, there are no reports of curcumin treatment-related toxicity in humans when administered at doses of up to 8 g/day for up to 3 months [41]. Additionally, more than 300 clinical trials have demonstrated the protective effects of curcumin against a variety of conditions, including respiratory, liver, inflammatory, and metabolic diseases [42,43]. Curcumin has shown good safety profiles, broad-spectrum antiviral activities, and protective effects in multiple organs, making it a promising candidate for complementary treatment of SARS-CoV-2 infection.

Curcumin represents an ideal scaffold for COVID-19 drug discovery, and impressive progress has been achieved in research associated with the development of curcumin-related anti-SARS-CoV-2 drugs [44,45]. For example, Bormann et al. [22] demonstrated that pure curcumin effectively neutralizes SARS-CoV-2 in Vero E6 cells with an EC_50_ of 21.2 µM. In the same study, curcumin reduced SARS-CoV-2 RNA levels in Vero E6 cells with an EC_50_ of 38 µM. Notably, both curcumin-containing nutritional supplement capsules and turmeric root extract are shown to completely neutralize SARS-CoV-2 in vitro. Marín-Palma et al. [46] reported that curcumin (10 µg/mL) exerted anti-SARS-CoV-2 effects of 99.0% and 99.8% against the DG614 strain and the Delta variant in Vero E6 cells, respectively. Mechanistic studies have revealed that curcumin can inactivate cellular enzymes involved in viral fusion with host membranes, thus blocking viral entry. Importantly, curcumin is shown to prevent the production and release of IL-1β, IL-6, MCP-1, and IL-8. Furthermore, Bahun et al. [47] reported that curcumin effectively inhibited SARS-CoV-2 M^pro^ replication in vitro (IC_50_ = 11.9 μM). Meanwhile, in silico molecular dynamics studies indicated that the M^pro^ active sites Gln192 and Arg188 participate in hydrogen bonding interactions with curcumin [47].

Several trials have tentatively investigated the therapeutic effect of curcumin (Figure 2b). For example, Ujjan et al. [48] conducted an open-label, randomized, controlled clinical trial (ClinicalTrials.gov: NCT04603690) in Pakistan to evaluate the efficacy and safety of an oral curcumin-quercetin supplement plus standard of care vs. standard of care alone in outpatients with early-stage, mild to moderately symptomatic COVID-19. In total, 50 patients were enrolled, 25 of which were assigned to receive a curcumin-quercetin supplement (daily intake of 168 mg of curcumin and 260 mg of quercetin, twice a day for 2 weeks) alongside standard of care, while the other 25 were assigned to receive only standard of care (control group). Viral clearance was significantly higher in the curcumin-quercetin group (18/25 [72.0%]) vs. 6/25 [24.0%], respectively; *p* = 0.0008). Furthermore, the percentage of acute COVID-19-associated symptoms was lower in the curcumin-quercetin group compared to the control group (complete symptom resolution: 40.0% [10/25] vs. 16.0% [4/25], respectively; *p* = 0.061). No treatment-emergent adverse effects were observed in the curcumin-quercetin group. Several other studies have obtained results consistent with these findings. Khan et al. [49] conducted another open-label, randomized, controlled trial (ClinicalTrials.gov: NCT05130671) in Pakistan to assess the efficacy of a 14-day treatment regimen comprising a daily oral co-supplementation of curcumin (168 mg), quercetin (260 mg), and vitamin D3 (9 µg) as adjuvant therapy in 25 patients with mild to moderate COVID-19. The authors reported that patients in the oral co-supplementation group exhibited accelerated negativization of the SARS-CoV-2 RT-PCR test compared to those in the control group (60.0% [15/25] vs. 20.0% [5/25], respectively; *p* = 0.009). Furthermore, Hellou et al. [50] conducted a double-blinded, multicenter, placebo-controlled Phase II clinical trial in Israel (ClinicalTrials.gov: NCT04382040) to evaluate the efficacy and safety of ArtemiC, an oral spray containing curcumin (40 mg), artemisinin (12 mg), frankincense (30 mg), and vitamin C (120 mg), for 15 days in hospitalized patients with symptomatic COVID-19 (*N* = 33 vs. *N* = 17 for the placebo group). The results showed that 91% of patients receiving ArtemiC oral spray displayed a significant improvement in the National Early Warning Score 2. Compared with the placebo, ArtemiC treatment shortened the duration of oxygen supplementation, hospital admission time, and abnormal oxygen saturation. Overall, accumulating clinical evidence supports the hypothesis that curcumin exerts beneficial effects in the treatment of COVID-19.

However, the clinical application of curcumin is greatly limited by its poor chemical stability and oral bioavailability, short half-life, and lack of target specificity [51,52]. These limitations may potentially be overcome via the application of nanotechnology (Figure 2c,d) and lead optimization (Figure 2e). For example, Gunathilake et al. [53] developed a curcumin-loaded, inhalable, nanotherapeutic (nanocellulose/polyvinyl alcohol/curcumin) for use in COVID-19. The curcumin in these nanoparticles had good water solubility (313.61 mg/L) and a high loading capacity (8.90 mg/g) and represented a promising alternative strategy for the treatment of COVID-19. De Maio et al. [54] fabricated personal protective equipment coated with graphene and curcumin. These coatings interacted with the SARS-CoV-2 surface, thereby trapping the virus and inhibiting further transmission. Importantly, the authors suggested that the combination of the mechanical and chemical actions of the two antimicrobials had the potential to limit the emergence and spread of resistant SARS-CoV-2 strains. Organic nanofibrous membranes with ultrafine particle filtration and high gas permeance show great potential in preventing SARS-CoV-2 infection [55]. Rao et al. [56] developed a biocompatible curcumin-incorporated composite membrane for pathogen sterilization and isolation via antibacterial-antiviral functionalization and controllable heating lamination. This filter exhibited excellent gas permeability (3423.6 m^3^/[m^2^·h·kPa]), a high ultrafine particle rejection rate (>98.79%), and good SARS-CoV-2 capture and kill efficiency (99.90% for 5 min). Moreover, the curcumin-incorporated filter also showed high antibacterial activity against a variety of bacteria (*Escherichia coli* [99.84%], *Bacillus subtilis* [99.02%]) and fungi (*Aspergillus niger* [93.60%], *Penicillium* [95.23%]) in vitro. More significantly, the filter showed good stability after 10 heating cycles, which was indicative of its reusability. This material could be widely used in the design of respirator masks. Moreover, Sharma et al. [57] prepared curcumin-encapsulated polysaccharide nanoparticles (Cur-PS-NPs) with monodisperse, spherical morphologies; diameters of 43 and 22 nm as measured using dynamic light scattering and transmission electron microscopy, respectively; a low polydispersity index (0.52); a high entrapment efficiency (25%); and excellent bioavailability. The Cur-PS-NPs attenuated the interaction between the angiotensin-converting enzyme 2 (ACE2) receptor and the SARS-CoV-2 S protein. Mechanistically, Cur-PS-NPs suppressed SARS-CoV-2 S protein-induced cytokine storms in liver Huh7.5 and lung A549 epithelial cells by inhibiting the NF-κB/MAPK signaling pathway, which in turn decreased the SARS-CoV-2 S protein-mediated phosphorylation of p38 MAPK, p42/44 MAPK, and p65/NF-κB, as well as the expression of p65/NF-κB. Importantly, treatment with Cur-PS-NPs had almost no effect on S protein-naive (unstimulated) Huh7.5 and A549 cells. The results of this study provided a foundation for the development of curcumin-based nanotherapeutics with improved curcumin bioavailability to treat COVID-19 by mitigating hyperinflammatory responses and preventing lung and liver injuries.

Lead optimization of curcumin provides another promising strategy for anti-SARS-CoV-2 drug development. For example, dimethoxycurcumin, a methylated analog of curcumin, can acidify endolysosomes and inhibit SARS-CoV-2 entry [58]. Compared to curcumin, dimethoxycurcumin has greater metabolic stability, as well as superior bioavailability and anti-inflammatory properties. (1E,6E)-1,7-Bis(4-(dimethylamino)phenyl)hepta-1,6-diene-3,5-dione (C-NMe2), a photodynamic dimethyl amino derivative of curcumin, exhibits high stability in an aqueous environment and improved interactions with two enzymes, which are important for SARS-CoV-2 replication, namely, PL^pro^ and ADP ribose phosphatase [59]. Dhaka et al. [60] revealed that bisdemethoxycurcumin, a natural demethoxy analog of curcumin, can effectively inhibit SARS-CoV-2 replication by targeting nucleocapsid protein with an EC_50_ value of 1.64 µM and with high selectivity (SI = 15.24).

These results support further clinical evaluation of curcumin as an alternative to existing targeted therapies for COVID-19. Current information on completed or ongoing clinical trials of curcumin for COVID-19 treatment is summarized in Table 1. Taken together, the above studies highlight that the delivery, efficacy, and bioavailability of curcumin-based COVID-19 therapies can be improved with the assistance of nanotechnology, combination therapies, and lead optimization.

## 3. Resveratrol and Its Natural Derivatives—Grape-Derived ACE2 Inhibitors That Can Limit SARS-CoV-2 Infection

**Figure 3:**
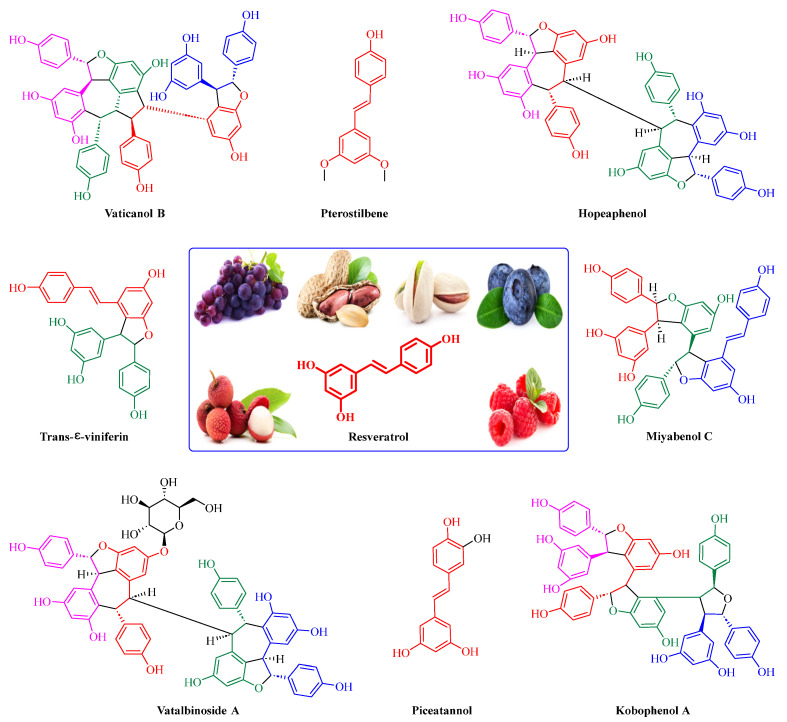
Chemical structure of resveratrol and its natural derivatives. Chemical structure of resveratrol, which can be isolated from several dietary sources such as grapes, peanuts, pistachios, blueberries, etc.; Chemical structures of piceatannol (hydroxylated analog) and pterostilbene (methoxylated analog); Chemical structures of trans-ε-viniferin (dimer), miyabenol C (trimer), and hopeaphenol (tetramer), vatalbinoside A (tetramer), vaticanol B (tetramer), and kobophenol A (tetramer).

Resveratrol (RSV), a polyphenolic nutraceutical mainly found in grapes (*Vitis vinifera* L.) (Figure 3), demonstrates health-promoting properties, including antioxidative [61], immunomodulatory [62], and anti-aging effects [63]. Since its association with the “French paradox” in 1992, resveratrol has received increasing research interest owing to its diverse pharmacological activities and multi-organ protective effects (e.g., acute lung injury [64], cerebral ischemia/reperfusion injury [65], myocardial cell apoptosis [66], and hepatic injury [67]). Resveratrol is classified as a food supplement with an excellent safety record and no serious adverse events even when administered in large doses (up to 600 mg per day) [68]. This nutraceutical is recognized as a promising therapeutic against a variety of viruses, including influenza A virus [69], respiratory syncytial virus [70], SARS-CoV [71], MERS-CoV [72], and HCoV-229E [73].

**Figure 4:**
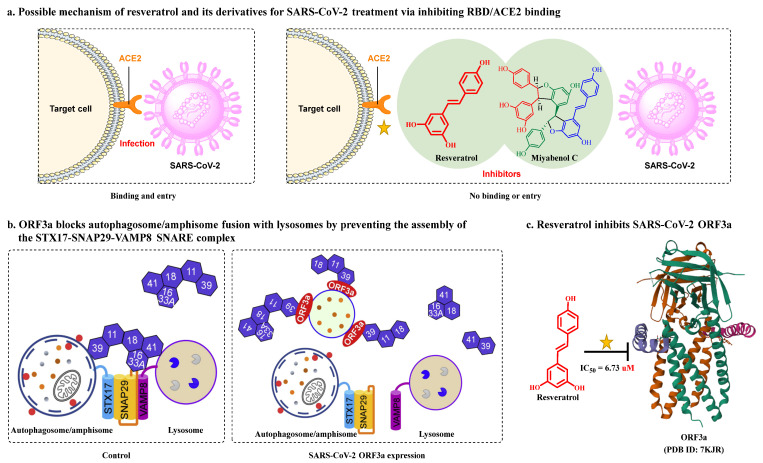
Possible mechanism of action of resveratrol and its derivatives for SARS-CoV-2 treatment. (**a**) Possible mechanism of action of resveratrol and its derivatives in the treatment of SARS-CoV-2 involving the inhibition of RBD/ACE2 binding. (**b**) A model showing how SARS-CoV-2 ORF3a impairs autophagosome maturation by disrupting the HOPS-mediated assembly of the SNARE complex. (Adapted with permission [75]. Copyright © 2020 Elsevier B.V.) (**c**) Resveratrol inhibits SARS-CoV-2 ORF3a. ORF3a, an essential contributor to infection and propagation of SARS-CoV-2, is a potential therapeutic target against COVID-19.

Numerous studies, both in vitro and in vivo, have shown that resveratrol is beneficial for SARS-CoV-2 treatment. Yang et al. [23] demonstrated that resveratrol significantly inhibits the replication of SARS-CoV-2 in Vero cells with an EC_50_ of 4.48 μM. Concomitantly, Pasquereau et al. [73] reported that resveratrol displays inhibitory activity against SARS-CoV-2 in Vero E6 cells with an EC_90_ and EC_50_ of 11.42 and 10.66 μM, respectively. Bahun et al. [47] found that resveratrol inhibited M^pro^ activity with an IC_50_ value of 16.9 µM. SARS-CoV-2 open reading frame-3a (ORF3a), an accessory protein implicated in autophagy inhibition, inflammasome activation, and apoptosis, is a potential therapeutic target against COVID-19 [74,75] (Figure 4b). Fam et al. [76] indicated that resveratrol shows promise as an ORF3a inhibitor, with an IC_50_ of 6.73 μM (Figure 4c). The post-acute sequelae of SARS-CoV-2 infection include long-term effects on tissues and organs [77]. Notably, adjunctive therapy with resveratrol helps reduce SARS-CoV-2 infection-induced inflammation, thereby improving patient outcomes. For example, in Brazil, de Souza Andrade et al. [78] evaluated the in vitro effect of resveratrol on neutrophil extracellular trap (NET) production in 190 hospitalized patients with moderate, severe, or critical COVID-19. The results clearly showed that resveratrol significantly attenuated NET formation and increased neutrophil viability in patients with severe disease. Idiopathic pulmonary fibrosis is a key feature in many post-COVID-19 patients [78]. Sheng et al. [79] demonstrated that piceatannol, a naturally occurring hydroxylated resveratrol analog, protects against bleomycin-induced pulmonary fibrosis by targeting the Smad3/ERK/p38 signaling pathway.

Combination therapy has been proposed as a potential strategy for treating SARS-CoV-2 infection [80]. Goc et al. [81,82] evaluated the in vitro efficacy of a specific combination of plant extracts and micronutrients (a mixture composed of resveratrol, curcumin, quercetin, baicalin, vitamin C, theaflavin, N-acetylcysteine, naringenin, and broccoli extract) against SARS-CoV-2 and its Alpha, Beta, Gamma, Delta, Kappa, Mu, and Omicron variants. They found that this combination exerted significant inhibitory effects against SARS-CoV-2 and its variants in vitro via pleiotropic mechanisms, including the targeting of viral RdRp, furin, and cathepsin L activity. At a concentration of 10 μg/mL, combination therapy of resveratrol with naturally occurring compounds enhanced treatment efficacy compared to a single compound (i.e., inhibited RBD binding to the human ACE2 receptor by 90% compared to the control treatment). These findings have important implications for ensuring the effective treatment of SARS-CoV-2 and emerging sarbecovirus clades [81,82]. Polydatin and pterostilbene, natural precursors of resveratrol, have stronger antioxidant effects and better bioavailability than resveratrol [83,84]. De Angelis et al. [85] reported that a mixture containing these polyphenols plus ellagic acid, honokiol, zinc, selenium, and chromium showed greater efficacy in inhibiting SARS-CoV-2 infection than polydatin or pterostilbene treatment alone.

Resveratrol oligomers (condensation ≥ 2), with unique structures and pleiotropic biological activities, have attracted substantial attention as potential food additives for COVID-19 treatment (Figure 3). A library composed of 512 compounds derived from natural products was screened using a high-throughput RBD/ACE2 binding assay and three resveratrol oligomers—hopeaphenol, vatalbinoside A, and vaticanol B—were identified as inhibiting both RBD/ACE2 binding (IC_50_ values of 0.11, 0.24, and 0.067 μM, respectively) and M^pro^ activity (IC_50_ values of 42.5, 68.7, and 47.6 μM, respectively) [86]. These results suggested that resveratrol oligomers are effective at inhibiting RBD/ACE2 binding (Figure 4a). Notably, no evidence of cytotoxicity was observed with these compounds. Additionally, Tietjen et al. [86] reported that hopeaphenol can inhibit the cellular entry of USA-WA1/2020, Alpha, and Beta variants of SARS-CoV-2 with EC_50_s of 23.4, 7.8, and 7.5 μM, respectively. These results further underscore the potential of these three resveratrol oligomers as attractive candidates for broad-spectrum antiviral therapy with the ability to respond to emerging SARS-CoV-2 variants. Gangadevi et al. [87] demonstrated that kobophenol A, a resveratrol tetramer, effectively blocks the interaction between the host ACE2 receptor and S1-RBD in vitro with an IC_50_ of 1.81 μM, and inhibits SARS-CoV-2 infection in VeroE6 cells with an EC_50_ of 71.6 μM. The protease cathepsin L is essential for SARS-CoV-2 infection [88]. Wang et al. [89] found that two resveratrol oligomers, miyabenol C (IC_50_ = 3.08 μM) and *trans*-ε-viniferin (IC_50_ = 40.4 μM), specifically inhibited the entry of SARS-CoV-2 by inhibiting cathepsin L activity. Resveratrol oligomers show a better pharmacokinetic profile than resveratrol itself. Accordingly, non-toxic, natural-product resveratrol oligomers may be promising lead compounds for COVID-19 treatment.

Resveratrol exhibits low bioavailability owing to its poor solubility and rapid metabolism and must be administered in very high oral doses to achieve therapeutic efficacy [90]. Given these critical limitations, efforts have been made in the field of nanomedicines to exploit other therapeutic agents capable of targeting SARS-CoV-2. Zakaria et al. [91] prepared spherical resveratrol-loaded nano-bilosomes (F5) with a mean diameter of 228.9 nm, a zeta potential of −39.8 mV, a high drug entrapment efficiency (86.1%), and superior cellular uptake (~4.7-fold greater than that for resveratrol in Caco-2 cells). F5 exhibited an IC_50_ of 0.24 μg/mL against SARS-CoV-2, a 6.6-fold improvement compared with an IC_50_ of 1.6 μg/mL for resveratrol. Interestingly, F5 has a good safety profile, with an SI of 139.5 for F5 and 2.9 for resveratrol. Importantly, F5 could overcome the extensive first-pass liver metabolism and degradation associated with oral resveratrol administration. This study provides a basis for the development of nanoparticles containing resveratrol as oral remedies for COVID-19.

## 4. Gossypol—A Cotton Plant-Derived RNA-Dependent RNA Polymerase Inhibitor with Broad-Spectrum Anti-Coronavirus Activity

RdRp, an essential therapeutic target that catalyzes the replication of RNA from RNA template, is highly conserved in positive-sense single-stranded RNA viruses, including SARS-CoV-2 [92]. Plant viruses cause devastating diseases in many important agriculture systems worldwide, yet studies show that *Gossypium* spp. (cotton plant) display strong resistance to single-stranded RNA viruses [93], which provide proactive drug design strategies to minimize the impact of antiviral drug resistance.

**Figure 5:**
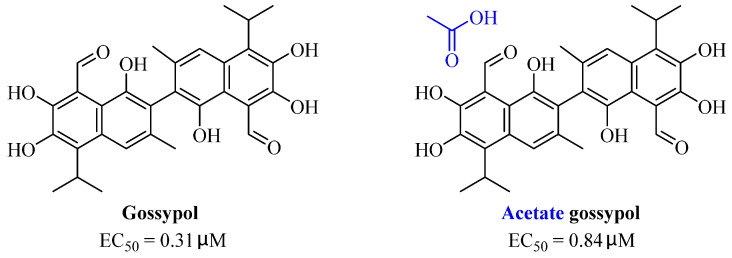
The chemical structures of gossypol and acetate gossypol.

An enantiomeric mixture (atropisomerism) of natural polyphenol gossypol (Figure 5), an extraction commonly derived from the cotton plant (stems, leaves, roots, bolls, and seeds) has been shown to exhibit antioxidant [94], antivirus [95], antimicrobial [96], and anticancer properties [97]. Gossypol (GOS) is reported to be effective at treating lung diseases, including non-small cell lung cancer [98], lung injury [99], and pulmonary fibrosis [100]. GOS exists as enantiomers due to the restricted rotation around the internal binaphthyl bond. Interestingly, (−)-gossypol [(−)-GOS] is shown to be active as a male contraceptive, whereas (+)-gossypol [(+)-GOS] has previously been reported to be inactive [101]. Furthermore, its main therapeutic form, gossypol acetate (GOSAc) (Figure 5), an equimolar crystalline complex of racemic gossypol with acetic acid, is a clinically approved gynecological drug used to treat uterine leiomyoma in China [102]. Wang et al. [24] demonstrated that GOS, a promising lead compound, is beneficial for SARS-CoV-2 treatment (Table 2). To be specific, firstly, an antiviral library consisting of 881 cotton natural compounds was screened initially in vitro as potential counters to SARS-CoV-2 infection. Among these candidates, GOS (IC_50_ = 14.15 μM), (−)-GOS (IC_50_ = 15.17 μM), and GOSAc (IC_50_ = 14.83 μM) were identified, displaying more potent inhibitory effects against SARS-CoV-2 RdRp than the nucleoside RdRp inhibitor, remdesivir triphosphate (IC_50_ = 37.67 μM), and the non-nucleoside RdRp inhibitor, baicalein (IC_50_ = 62.55 μM), in vitro. In addition, GOS, GOSAc, and (−)-GOS were also effective dose-dependent inhibitors of SARS-CoV-2 replication in vitro in Vero E6 cells with EC_50_ values of 0.31 μM, 0.84 μM, and 0.72 μM, respectively, and no significant cellular cytotoxicity. In addition, the therapeutic indexes were 116.71, 42.17, and 61.82, respectively, suggesting that the antiviral effect of GOS was not affected by its optical activity [24].

**Figure 6:**
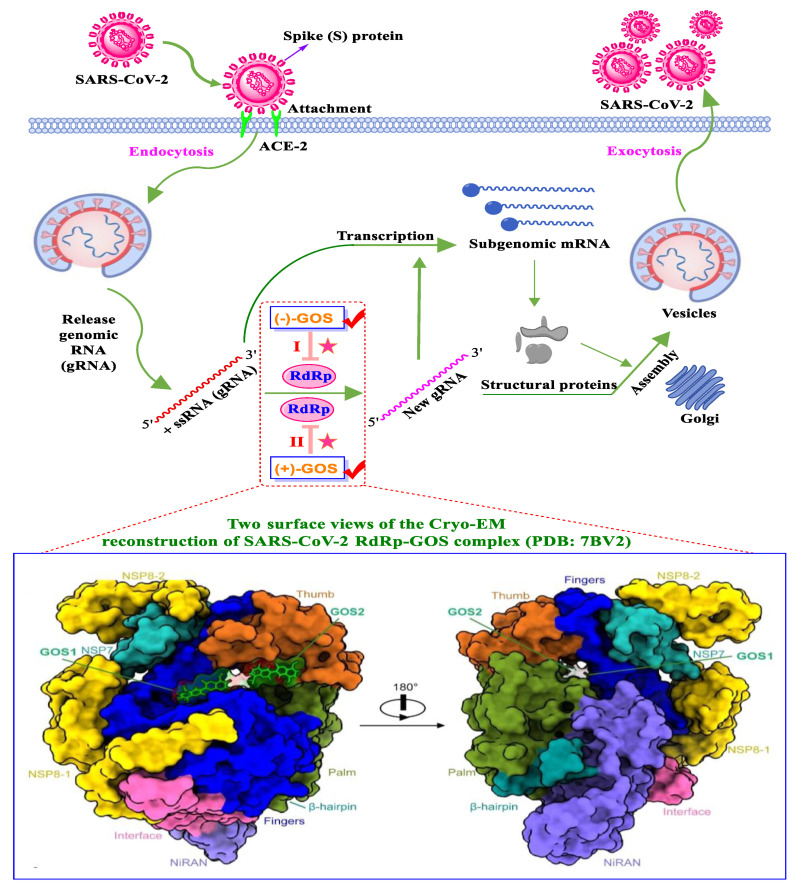
Schematic illustration of two gossypol (GOS) molecules acting as SARS-CoV-2 inhibitors via blocking the highly conserved RNA-dependent RNA polymerase. GOS inhibits RdRp activity by occupying the binding site for the RNA template [(−)-GOS, named GOS1] and primer [(+)-GOS, named GOS2]), consequentially inhibiting the catalytic activity of RdRp [24].

Additionally, Wang et al. [24] revealed that GOS administered via the intramuscular or intranasal route can enhance anti-SARS-CoV-2 activity in a mouse model, resulting in significantly reduced SARS-CoV-2 replication in nasal turbinate in a dose-dependent manner. To elucidate the underlying inhibitory mechanism at a molecular level, the authors [24] determined the 3.36 Å crystal structure of GOS in complex with SARS-CoV-2 RdRp (PDB ID: 7BV2). This structure indicates that two GOS molecules ([(−)-GOS] and [(+)-GOS]) occupy the center of the central cavity and jointly reduce the cavity opening size (Figure 6). Further analysis of the complex structure revealed that GOS inhibits RdRp activity by occupying the binding site for the RNA template [(−)-GOS] and primer [(+)-GOS]), consequentially inhibiting the catalytic activity of RdRp (Figure 6). Specifically, in vitro, data suggest that RdRp mutants of SARS-CoV-2 variants (including Omicron, the RdRpP323L mutation [IC_50_ = 11.37 μM]; Delta, RdRpP323L; G671S mutation [IC_50_ = 13.23 μM]) remain susceptible to GOS [24]. Furthermore, GOS exerted significant suppressive effects on the SARS-CoV-2 Delta variant in Vero E6 cells (EC_50_ = 0.23 μM, SI = 157.3). In addition to SARS-CoV-2, GOS exhibits broad-spectrum antiviral effects in vitro, including against porcine epidemic diarrhea virus (PEDV, alpha-CoV, EC_50_ = 0.99 μM, SI = 36.55), SADS-CoV (alpha-CoV, EC_50_ = 2.55 μM, SI = 14.19), infectious bronchitis virus (IBV, gamma-CoV, EC_50_ = 1.02 μM, SI = 35.47), and porcine deltacoronavirus (PDCoV, delta-CoV, EC_50_ = 1.06 μM, SI = 19.35), suggesting that GOS can act as a pan-coronavirus inhibitor [24]. A molecular docking model revealed that GOS can recognize diverse coronaviruses by targeting the highly conserved RdRp. The binding energies of GOS with RdRps of PEDV, SADS-CoV, IBV, and PDCoV were −7.6, −8.4, −7.9, and 8.8 kcal/mol, respectively [24].

It is important to note that high concentrations of GOS may cause several side effects, including respiratory distress [103]. As the lung is the primary target tissue of SARS-CoV-2, maintaining an effective lung concentration of antiviral drugs is required. Previous studies have demonstrated that cells are more sensitive to (−)-GOS. Considering that the antiviral effect of GOS were not affected by its optical activity, the development of (+)-GOS-derived antiviral drugs may be promising for further lead optimization.

## 5. Other Natural Dietary Polyphenols Anti-SARS-CoV-2 Candidates in Development

Numerous studies, both in in vitro and in vivo, have shown that curcumin, resveratrol, and gossypol are beneficial for COVID-19 treatment via targeting of M^pro^, the S protein, and RdRp of SARS-CoV-2 and its variants. In addition, other dietary polyphenols have shown promising potential for SARS-CoV-2 treatment and prevention that provide more options for controlling COVID-19 infection. As shown in Table 3, these polyphenols can inhibit SARS-CoV-2 replication and demonstrate strong in vitro activity, but additional in-depth research and further optimization are still required.

## 6. Conclusions and Future Directions

The COVID-19 pandemic highlights the need for novel strategies to rapidly develop therapies and vaccines against emerging viral diseases. Natural dietary polyphenols (e.g., curcumin, resveratrol, and gossypol) are essential complements to vaccines and small-molecule therapies and have significant potential for use in the prevention and therapy of SARS-CoV-2 and emerging variants. However, some limitations (e.g., poor stability, weak oral bioavailability, short half-life, and lack of target specificity) cannot be ignored. Multiple measures must be considered if dietary polyphenols are to be used to provide broad-spectrum or universal protection against emerging variants.

First, developing personal protective equipment (e.g., reusable, antiviral, and antibacterial masks) containing natural dietary polyphenols is a promising inexpensive and convenient option for preparing for future pandemics. For example, De Maio et al. [54] fabricated personal protective equipment coated with graphene and curcumin. This graphene/curcumin-based antiviral coating interacted with the SARS-CoV-2 surface, trapping the virus, thus preventing further transmission. Notably, the combination of two antimicrobials could limit the emergence and spread of resistant SARS-CoV-2 strains. In parallel, Rao et al. [56] developed a biocompatible curcumin-incorporated composite membrane that exhibited excellent gas permeability (3423.6 m^3^/[m^2^·h·kPa]), a high ultrafine particle rejection rate (>98.79%), and good SARS-CoV-2 capture and kill efficiency (99.9% for 5 min). The identification of efficient ways to deliver polyphenols to the site of infection (mainly targets respiratory cells) is critical for future drug development. Optimized delivery devices provide new options for delivering dietary anti-SARS-CoV-2 polyphenols.

Second, several measures (e.g., nanotechnology, combination therapies, and lead optimization) must be considered to develop dietary polyphenol-based next-generation therapeutic agents with improved pharmacokinetic, resistance, and adverse event profiles. For example, spherical resveratrol-loaded nano-bilosomes (F5) exhibited a higher activity (IC_50_ of 0.24 μg/mL for F5 and 1.6 μg/mL for resveratrol) and an improved safety profile (SI of 139.5 for F5 and 2.9 for resveratrol) [91]. The availability of curcumin-loaded inhalable nanocellulose (nanocellulose/polyvinyl alcohol/curcumin), with its improved water solubility (313.61 mg/L) and high loading capacity (8.90 mg/g) is also key for future development [53]. Importantly, inhaled nanomedicines should be made with food-grade materials and should not affect normal respiratory functions. Regarding combination therapies, the literature shows that a specific combination of plant extracts and micronutrients (including resveratrol, curcumin, quercetin, vitamin C, theaflavin, naringenin, and broccoli extract) exert significant inhibitory effects against SARS-CoV-2 and its Alpha, Beta, Gamma, Delta, Kappa, Mu, and Omicron variants in vitro via pleiotropic mechanisms, including the targeting of viral RdRp, furin, and cathepsin L activity [81,82]. Lead optimization provides another promising strategy for further drug development. For instance, bisdemethoxycurcumin, a natural demethoxy analog of curcumin, can effectively inhibit SARS-CoV-2 replication by targeting nucleocapsid protein with an EC_50_ value of 1.64 µM and with high selectivity (SI = 15.24) [60]. Nanotechnology, combination therapies, and lead optimization may be potential strategies to solve this problem, but further research and optimization are required.

Third, more validation studies with high-quality clinical data for natural dietary polyphenols are urgently needed to assess their effects and potential. Although some preliminary clinical trial results have demonstrated their potential, the sample sizes are small, and further validation is required. We must gain an improved understanding of the metabolism of dietary polyphenols in the human body and further evaluate the potential risks that may arise from their use. At the same time, individual differences, dose adjustments, and possible adverse reactions should also be considered in clinical trials. Nonetheless, we believe that dietary polyphenols can be regarded as a first-line option for managing SARS-CoV-2 infection via well-formulated in-depth research and rigorous monitoring.

## Figures and Tables

**Table 1:** Curcumin-related clinical trials based on a systematic search of ClinicalTrials.gov (https://clinicaltrials.gov/, accessed 15 August 2023).

Interventions	Principal Investigator	Identifier (Year)	Participants	Details
Curcumin, quercetin, and vitamin D3	Liaquat University Hospital Sindh, Pakistan	NCT04603690 (2020)	50	Oral curcumin, quercetin, and vitamin D3 supplements for mild to moderate symptoms of COVID-19
Micellar ArtemiC, comprising curcumin, artemisinin, frankincense, and vitamin C	MGC Pharmaceuticals d.o.o	NCT04382040 (2020)	50	A phase II, controlled clinical study designed to evaluate the effect of ArtemiC in COVID-19 patients
Curcumin and palmitoylethanolamide	Arizona Biomedical Collaborative Arizona, United States	NCT04912921 (2021)	115	Effect of palmitoylethanolamide on proinflammatory markers in adults diagnosed with COVID-19
Curcumin and quercetin	King Edward Medical University, Punjab, Pakistan	NCT05130671 (2021)	50	Nutritional supplementation of quercetin and curcumin for early mild symptoms of COVID-19
Nanoparticular CimertrA, comprising curcumin, artemisinin, boswellia, and vitamin C	MGC Pharmaceuticals d.o.o	NCT04802382 (2021)	252	A phase III clinical study to evaluate the effect of CimetrA in patients diagnosed with COVID-19
Curcumin, boswellia serrata, and ascorbic acid	PhysioMetrics	NCT05150782 (2021)	32	To examine the effect of a mixture of micellized curcumin, boswellia serrata, and ascorbic acid on patients with long COVID
Nanoparticular CimertA, comprising curcumin, boswellia, and vitamin C	MGC Pharmaceuticals d.o.o	NCT05037162 (2021)	240	A phase II, multi-center study in Israel, Brazil, Spain, and South Africa to evaluate the effect of CimetrA on COVID-19 patients
NASAFYTOL^®^ Capsules, containing curcumin, turmeric extract, quercetin, and vitamin D3	Tilman S.A.	NCT04844658 (2021)	51	To evaluate the effect and safety of NASAFYTOL^®^ on COVID-19-positive hospitalized patients
Nutritional powder (including Curcumin)	Shanghai Tongji Hospital, Tongji University School of Medicine, China	NCT05629975 (2022)	150	Oral nutritional supplements in the treatment of elderly mild to moderate COVID-19

**Table 2:** Broad-spectrum antiviral activity of gossypol and related derivatives in vitro.

Compound	Virus	EC_50_ or IC_50_ (μM)	Therapeutic Indexes
Gossypol (GOS)	SARS-CoV-2	IC_50_ = 14.15 (RdRp)	-
		EC_50_ = 0.31 (Vero E6 cells)	116.71
		EC_50_ = 0.76 (Calu-3 cells)	52.07
	SARS-CoV-2 Delta variant (RdRp^P323L; G671S^ mutation)	IC_50_ = 13.23 μM (RdRp)	-
		EC_50_ = 0.23 (Vero E6 cells)	157.3
	SARS-CoV-2 Omicron variant (RdRp^P323L^ mutation)	IC_50_ = 11.37 (RdRp)	-
	PEDV	EC_50_ = 0.99 (Vero E6 cells)	36.55
	SADS-CoV	EC_50_ = 2.55 (Vero E6 cells)	14.19
	IBV	EC_50_ = 1.02 (Vero E6 cells)	35.47
	PDCoV	EC_50_ = 1.06 (Vero E6 cells)	19.35
(−)-GOS	SARS-CoV-2	IC_50_ = 15.17 (RdRp)	-
		EC_50_ = 0.84 (Vero E6 cells)	42.17
GOSAc	SARS-CoV-2	IC_50_ = 14.83 (RdRp)	-
		EC_50_ = 0.72 (Vero E6 cells)	61.82

**Table 3:** Other natural dietary polyphenols for treating SARS-CoV-2 infection in vitro.

Name	Species	Structure	EC_50_ or IC_50_ (μM)	Target or Mechanism	Refs.
Licochalcone B	*Glycyrrhiza uralensis* Fisch	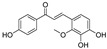	15.53	M^pro^	[104,105]
Echinatin	*Glycyrrhiza inflata*	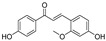	7.86	M^pro^	[104,105]
Neferine	*Nelumbinis plumula*	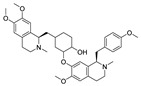	0.36	Inhibiting Ca^2+^-dependent membrane fusion and suppressing virus entry; RdRp	[106,107]
Salvianolic acid A	*Salvia miltiorrhiza*	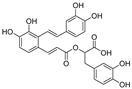	2.49	M^pro^	[108,109,110]
Corilagin	*Phmllanthi Fructus*	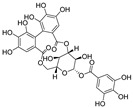	0.13	RBD-ACE2	[111,112,113]
Ellagic acid	*Punica granatum*	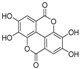	11.8	RBD-ACE2, M^pro^	[114,115]
Punicalagin	*Punica granatum*	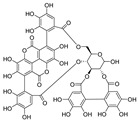	6.19	M^pro^	[116]
Chebulagic acid	*Terminalia chebula Retz*	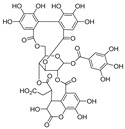	9.76	M^pro^	[116,117,118]
Epicatechin-3-*O*-gallate	*Camellia sinensis* var. *sinensis*	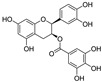	5.21	M^pro^	[119,120]
Catechin-3-*O*-gallate	*Senegalia catechu*	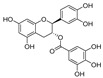	2.98	M^pro^	[119]
Hypocrellin A	*Hypocrella bambusae*	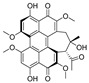	0.038	S protein	[121]
Binaphthoquinone	*Hypocrella bambusae*	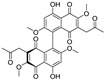	0.17	S protein	[121]
Shiraiachrome A	*Hypocrella bambusae*	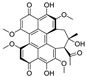	0.12	S protein	[121]
(+)-Shikonin	*Lithospermum erythrorhizon*	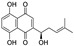	4.38	M^pro^	[122]
Shikonin	*Lithospermum erythrorhizon*	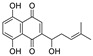	15.75	M^pro^	[123,124,125]
Phillyrin	*Forsythia suspensa*	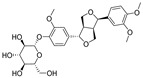	1.13	Inhibiting virus proliferation	[126,127]
Isoforsythiaside	*Forsythia suspensa*	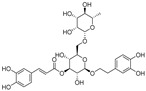	5.85	M^pro^	[128]
Forsythoside A	*Forsythia suspensa*	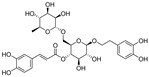	3.18	M^pro^	[128,129]
Forsythoside B	*Forsythia suspensa*	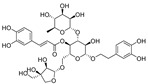	2.88	M^pro^	[128]
Acteoside	*Lippia triphylla*	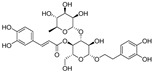	0.043	M^pro^	[130]
Panduratin A	*Boesenbergia rotunda*	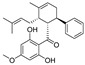	0.078	M^pro^	[131,132]
etc-pyrrolidinone C and D	*Camellia sinensis*	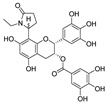	0.90	M^pro^	[133]
PGHG	*Penthorum chinense* Pursh	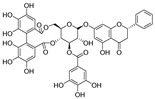	6.5	M^pro^, protein disulfide isomerase	[134]
Hypericin	*Hypericum perforatum* L.	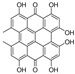	20.3	M^pro^	[135,136]
Theaflavin 3-gallate	black tea	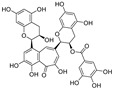	18.48	M^pro^, S protein	[137,138]
Theaflavin	black tea	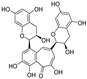	22.22	M^pro^	[137]
3,5-Dicaffeoylquinic acid	*Helichrysum bracteatum*	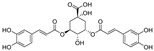	4.74	M^pro^	[139,140]
simplexoside (piperitol-O-β-D-glucoside)	*Helichrysum bracteatum*	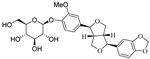	0.610	M^pro^	[139]
Geraniin	*Caryocar brasiliense*	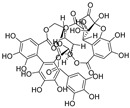	4.2	M^pro^, RBD-ACE2	[141,142,143]
Salvianolic acid A	*Salvia miltiorrhiza*	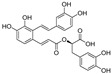	11.31	RBD-ACE2, M^pro^	[144,145]
Salvianolic acid B	*Salvia miltiorrhiza*	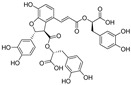	6.22	RBD-ACE2, M^pro^	[144,146]
Salvianolic acid C	*Salvia miltiorrhiza*	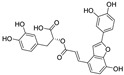	10.14	RBD-ACE2	[144,147]
Rosmarinic acid	*Salvia miltiorrhiza*	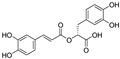	9.43	S protein, M^pro^	[148,149]
Liensinine	*Nelumbo nucifera* Gaertn.	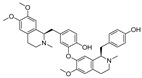	1.00	M^pro^	[150,151]
Forsythoside I	*Forsythia suspensa*	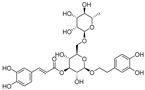	5.47	M^pro^	[128]
Forsythoside H	*Forsythia suspensa*	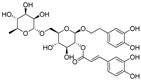	10.17	M^pro^	[128]
1,2,3,4,6-pentagalloylglucose (PGG)	*Toona sinensis*	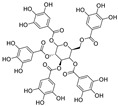	3.90	M^pro^	[152,153]
Epicatechin gallate	*Fagopyrum esculentum*	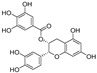	12.5	M^pro^	[154,155]
Mulberrofuran G	*Bombyx mori* L.	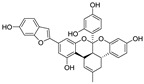	1.55	S Protein, M^pro^	[156,157]
Glabridin	*Glycyrrhiza glabra*	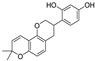	2.5	M^pro^	[158,159]
Dieckol	*Eisenia bicyclis*	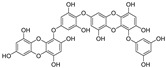	4.5	M^pro^, S protein	[160,161]
Tannic acid	*Galla chinensis*	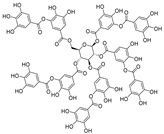	2.31	M^pro^, TMPRSS2	[162,163]

## Data Availability

No data were used for the research described in the article.
